# Should the NHS be privatized? Annual varsity medical debate - London, 22 January 2010

**DOI:** 10.1186/1747-5341-5-7

**Published:** 2010-05-11

**Authors:** Myura Nagendran, Sanjay Budhdeo, Mahiben Maruthappu, Kapil Sugand

**Affiliations:** 1 Emmanuel College, University of Cambridge, St Andrew's Street, Cambridge, CB2 3AP, UK; 2 New College, University of Oxford, Holywell Street, Oxford, OX1 3BN, UK; 3 Green Templeton College, University of Oxford, Woodstock Road, Oxford, OX2 6HG, UK; 4 School of Medicine, Imperial College London, South Kensington, London, SW7 2AZ, UK

## Abstract

The Varsity Medical Debate, between Oxford and Cambridge Universities, brings together practitioners and the public, professors, pupils and members of the polis, to facilitate discussion about ethics and policy within healthcare. The motion on privatizing the National Health Service (NHS) was specifically chosen to reflect the growing sentiment in the UK where further discourse upon models of healthcare was required. Time and again, the outcome of British elections pivots upon the topic of financial sustainability of the NHS. Having recently celebrated its sixtieth anniversary, the NHS has become heavily politicized in recent months, especially in the aftermath of the devastating global recession.

## Background

The 2010 Varsity Medical Debate between historical rivals Cambridge and Oxford Universities took place at the Royal College of General Practitioners in London on the 22^nd ^January. The debate was overseen by a prestigious panel of judges including the President of the College, Dr Iona Heath, and the former President of the Royal Society of Medicine, Baroness Finlay of Llandaff. Representatives from Oxford University proposed the motion that "This House would Privatize the Provision of Healthcare" and put forward the case for a privatized model, built upon an amalgamation of successful international frameworks (as seen in *Appendix 1 *[1]), to replace the secondary and tertiary care sectors in the ageing NHS. The motion was opposed by representatives from Cambridge University who argued that improving the publicly funded status quo was still plausible and preferable.

Since 1997, NHS expenditure has more than doubled from £35 billion to £89 billion [2]. However, a soaring deficit estimated at £3 billion per year has prompted many to question how much longer the current socialized system can be sustained. The 2006 Herceptin scandal is an example of the sensitive nature of any discussion of healthcare financing issues. Initially chemotherapy for breast cancer using this agent was not approved by the National Institute of Clinical Excellence (NICE) for public access due to its high cost. However, patient appeals to the High Court compelled NICE to reverse its initial decision [3]. Future controversy is undoubtedly inevitable as the NICE threshold of £20-30,000 per Quality-Adjusted Life Year (QALY) gained is tested by an ever more expensive array of drugs [4]. Other recent examples include the arthritis drug RoActemra and the cancer drug Nexavar; both of which are currently unavailable in England on grounds of unqualified cost-effectiveness: although an appeal against the Nexavar ruling is set to take place later this month [5].

Many argue that we are already proceeding toward a semi-privatized model with Private Finance Initiative (PFI) based hospitals and encroachment of service provision by Independent Sector Treatment Centres (ISTC), thereby blurring the line between private and governmental funding. It was in this context that the 2010 debate engaged a range of viewpoints on healthcare privatization. The issues discussed in the debate were delineated across key themes including economics, patient choice and ethics.

## Proposition's Privatized Prototype

Oxford proposed a model in which the role of the state as a regulator of care was distinguished from its role as a provider. An income tax-funded voucher scheme based upon a model suggested in the USA [6] would be used to purchase health insurance. Private companies could then competitively offer a variety of packages to suit individual needs. The basic state package would cost the minimum voucher price and patients would have the choice of increasing payment coverage to access additional services, such as private diagnostic scans. Crucially, the state would provide risk equalization subsidies to insurance companies to ensure competitive uptake of high-risk patients. Hospitals would be privately run and reputable companies would benefit from franchising to provide sub-specialty care. Primary care and acute emergency services would nevertheless remain state-provided.

## Economic and political implications

The Oxford team began their proposition by highlighting a range of factors that they believed made the current NHS unsustainable. Chief among these was increasing costs amidst decreasing efficiency. Oxford insisted that with a national debt of over £178 billion [7], crippling real term cuts to the current healthcare system were simply unavoidable.

Oxford claimed that their model would reduce costs and increase quality of care. This rested heavily on the belief that market forces and competition would significantly encourage efficiency. It was also stated that current government targets skew incentives so that arbitrary goals are met at the expense of quality of care. In the proposed model, risk equalization subsidies and competition between hospitals for patients would produce favorable clinical outcomes.

Cambridge strongly objected to the labeling of existing targets as arbitrary. They claimed that such targets did accurately reflect clinical outcomes and illustrated their point using the example of preventative blood pressure checks to ensure that patients at risk of heart attack and stroke are prophylactically maintained on statins. Furthermore, a comparison was made to the US' private healthcare system, where health indicators such as infant mortality are worse than in the UK, despite a significantly increased per capita expenditure [8]. Cambridge argued that reduced cost was neither an inherent nor guaranteed benefit of private healthcare.

## Patient Choice

A central tenet of the Oxford model was the enhanced fairness that would result from greater patient choice. Their reasons for this premise were two-fold: first, the availability of private healthcare separate from NHS treatment suggested that British society had accepted that those who earned more could purchase better care.

Moreover, they believed that there was a philosophical justification for this, in that removing the advantages of wealth removes the point of accumulating wealth, and thus removes productivity incentives in a capitalist society. Second, rather than just NHS and private options top-ups allow greater flexibility and accessibility to those who want to increase the quality of care according to both their health priorities and their earnings.

Oxford also highlighted that by choosing their healthcare packages, patients would assume more responsibility over their personal well-being. The very act of choosing insurance would not only compel them to consider the extent of health care afforded, but would also empower patients to recognize the limitations imposed by and upon their lifestyle. Additionally, the payment of premiums could incentivize patients to deter those lifestyle choices that have a negative impact on health (such as smoking and high-cholesterol diets).

Cambridge responded by asserting that increased choice, while beneficial, should not come at the heavy cost of access to healthcare. They claimed that lower social classes would have reduced availability to more complex and expensive interventions that require unaffordable top-ups. In addition, financially incentivizing patients to change their health behaviors could have a disastrous impact on chronic disease management. In such cases, patients would delay seeking medical advice, and this would lead to exacerbation of existing pathology, worsening of signs and symptoms, poorer prognoses, and eventually increased expenditure. Ultimately, the patients' access to healthcare provision should not be determined by their ability to afford it.

## Ethical considerations

A fundamental aspect of the Cambridge counter-argument was to identify access to healthcare as a basic human right. If a population is concerned about securing the civil liberties of all citizens, then a commitment should be made to ensure that the quality of life of the individual is adequate to make most of these liberties. Healthcare is instrumental, if not crucial, to quality of life. Cambridge was suspicious of the motives adopted by private companies, working from the presumption that if the *raison d'être *of these companies was to maximize profit, then quality and fairness would improve only for as long as financial gain was forecasted.

Oxford contended that even if healthcare was a right, it could be delivered more efficiently by the market and illustrated this by a comparison with the food industry; a clear example of market forces operating to increase efficiency and to keep food prices consistently low, while catering to consumer choice(s). Rest assured, no one attempted to compare patients to produce, yet the potential benefits evoked by the ideology of greater choice were well depicted.

## Conclusion

After much deliberation, the judges narrowly awarded victory to Oxford. Dr Heath praised the effort and debating skill of all participants and reiterated the importance of these discussions for the future of the NHS. A key point that was raised throughout the debate was the allocation of limited national resources. Cost, access and quality of care are all vital, but improvements in one factor may come at the expense of another. Oxford's view was that decreasing access to non-essential care services for a small minority would result in a disproportionate benefit in cost, access and quality for the majority. Cambridge's view was that access was paramount. Ultimately, what is important are the values and views of society. Surveys consistently show that the British public are still overwhelmingly in favor of free health service and echo the 'from cradle to grave' philosophy, or rather yet 'from womb to tomb' nowadays, of healthcare provision. Nevertheless, it is clear that crucial changes and stricter legislation will be required in the coming years. Both the administrative and financial infrastructure of the NHS must be sustainable in times of economic and political flux if the system is to cope with the needs and demands of an aging population, greater immigration and ever more costly market.

It was encouraging to see both the enthusiasm of the multi-disciplinary audience members, and versatility of the debaters in critically discussing and engaging controversial issues despite the relatively early stage of their professional training. In the end, it was unanimously agreed that today's students will ultimately have a most difficult, and the most crucial role in shaping tomorrow's NHS.

## Appendix 1

Modern healthcare delivery is mainly categorized in the following four sectors [1]:

A. Socialized medicine (e.g. UK) which is funded by the state.

B. Socialized insurance (e.g. France) which provides services for fees.

C. Mandatory insurance (e.g. Germany) which relies on multiple sickness funds.

D. Voluntary insurance (e.g. USA) which depends on private and commercialized companies offering services for fees through various packages.

## Conflict of interests

The authors declare that they have no competing interests.

## Authors' contributions

MN and SB organized the conference. MM and SB founded the event. KS compiled, formatted and submitted the manuscript. All authors have equally contributed, read and approved the final manuscript.
